# Supplementary material for: AurkA nuclear localization is promoted by TPX2 and counteracted by protein degradation
Source: Life Sci Alliance. 2023 Feb 16;6(5):e202201726. doi: 10.26508/lsa.202201726 (PMC9936162; doi:10.26508/lsa.202201726)
Supplement: Supplementary file 1 [file LSA-2022-01726_TableS1.docx]

| **Type** | **Description** | **Samples** | ***AURKA*** | | ***TPX2*** | |
| --- | --- | --- | --- | --- | --- | --- |
|  |  |  | ***Correlation*** | ***p-value*** | ***Correlation*** | ***p-value*** |
|  |  |  |  |  |  |  |
| **ACC** | Adrenocortical carcinoma | 77 | 0.80 | 1.6e−18 | 0.79 | 2.8e−17 |
| **BLCA** | Bladder Urothelial Carcinoma | 404 | 0.81 | 1.2e−93 | 0.74 | 1.2e−70 |
| **BRCA** | **Breast invasive carcinoma** | **1085** | **0.82** | **4e−303** | **0.69** | **2.2e−152** |
| **CESC** | Cervical squamous cell carcinoma | 306 | 0.68 | 4.2e−43 | 0.59 | 9.6e−30 |
| **COAD** | Colon adenocarcinoma | 275 | 0.80 | 3.1e−63 | 0.84 | 7.9e−73 |
| **ESCA** | Esophageal carcinoma | 182 | 0.67 | 2.5e−25 | 0.8 | 3.3e−41 |
| **GBM** | Glioblastoma multiforme | 163 | 0.75 | 1.9e−30 | 0.78 | 5.4e−35 |
| **HNSC** | Head and Neck squamous cell carcinoma | 519 | 0.76 | 3.1e−100 | 0.7 | 9e−79 |
| **KICH** | Kidney Chromophobe | 66 | 0.52 | 1.2e−05 | 0.45 | 0.00017 |
| **KIRC** | Kidney renal clear cell carcinoma | 523 | 0.43 | 8.1e−25 | 0.37 | 2.4e−18 |
| **KIRP** | Kidney renal papillary cell carcinoma | 286 | 0.61 | 2.1e−30 | 0.54 | 7.5e−23 |
| **LAML** | Acute Myeloid Leukemia | 173 | 0.59 | 2.2e−17 | 0.61 | 3.8e−19 |
| **LGG** | Brain Lower Grade Glioma | 518 | 0.58 | 3e−48 | 0.58 | 4.5e−47 |
| **LIHC** | Liver hepatocellular carcinoma | 369 | 0.64 | 4.3e−44 | 0.71 | 1.2e−58 |
| **LUAD** | Lung adenocarcinoma | 483 | 0.71 | 5.3e−76 | 0.72 | 2e−79 |
| **LUSC** | Lung squamous cell carcinoma | 486 | 0.75 | 4.6e−89 | 0.62 | 3.4e−53 |
| **MESO** | Mesothelioma | 87 | 0.73 | 1.2e−15 | 0.69 | 8.3e−14 |
| **OV** | Ovarian serous cystadenocarcinoma | 426 | 0.69 | 1.7e−60 | 0.6 | 1.8e−43 |
| **PAAD** | Pancreatic adenocarcinoma | 179 | 0.70 | 5.8e−28 | 0.67 | 1.3e−24 |
| **PCPG** | Pheochromocytoma and Paraganglioma | 182 | 0.44 | 3.4e−10 | 0.42 | 2.4e−09 |
| **PRAD** | Prostate adenocarcinoma | 492 | 0.62 | 1.9e−54 | 0.59 | 2.5e−47 |
| **READ** | Rectum adenocarcinoma | 92 | 0.75 | 3.8e−18 | 0.77 | 3.8e−19 |
| **SARC** | Sarcoma | 262 | 0.74 | 3.4e−46 | 0.69 | 4.7e−39 |
| **SKCM** | Skin Cutaneous Melanoma | 461 | 0.71 | 7.9e−71 | 0.73 | 7e−77 |
| **STAD** | Stomach adenocarcinoma | 408 | 0.75 | 6.8e−74 | 0.81 | 6.4e−96 |
| **TGCT** | Testicular Germ Cell Tumors | 137 | 0.66 | 3.6e−18 | 0.51 | 2.2e−10 |
| **THCA** | Thyroid carcinoma | 512 | 0.30 | 2.8e−12 | 0.29 | 9.9e−12 |
| **THYM** | Thymoma | 118 | 0.56 | 3.9e−11 | 0.48 | 4.5e−08 |
| **UCEC** | Uterine Corpus Endometrial Carcinoma | 174 | 0.82 | 7.2e−45 | 0.74 | 1.4e−31 |
| **UCS** | Uterine Carcinosarcoma | 57 | 0.68 | 6.8e−09 | 0.53 | 2.1e−05 |
| **UVM** | Uveal Melanoma | 79 | 0.66 | 5.5e−11 | 0.49 | 3.9e−06 |

**Supplementary Table 1**. Spearman Correlation analysis of CSE1L with AurkA and TPX2 in multiple cancer types (Tissue Samples > 50). Breast Invasive Carcinoma, with the highest p-value, is highlighted.
